# BERTtoCNN: Similarity-preserving enhanced knowledge distillation for stance detection

**DOI:** 10.1371/journal.pone.0257130

**Published:** 2021-09-10

**Authors:** Yang Li, Yuqing Sun, Nana Zhu

**Affiliations:** 1 College of Information and Computer Engineering, Northeast Forestry University, Harbin, Heilongjiang, China; 2 Library, Harbin University, Harbin, Heilongjiang, China; 3 School of Information Management, Heilongjiang University, Harbin, Heilongjiang, China; National University of Singapore, SINGAPORE

## Abstract

In recent years, text sentiment analysis has attracted wide attention, and promoted the rise and development of stance detection research. The purpose of stance detection is to determine the author’s stance (favor or against) towards a specific target or proposition in the text. Pre-trained language models like BERT have been proven to perform well in this task. However, in many reality scenes, they are usually very expensive in computation, because such heavy models are difficult to implement with limited resources. To improve the efficiency while ensuring the performance, we propose a knowledge distillation model BERTtoCNN, which combines the classic distillation loss and similarity-preserving loss in a joint knowledge distillation framework. On the one hand, BERTtoCNN provides an efficient distillation process to train a novel ‘student’ CNN structure from a much larger ‘teacher’ language model BERT. On the other hand, based on the similarity-preserving loss function, BERTtoCNN guides the training of a student network, so that input pairs with similar (dissimilar) activation in the teacher network have similar (dissimilar) activation in the student network. We conduct experiments and test the proposed model on the open Chinese and English stance detection datasets. The experimental results show that our model outperforms the competitive baseline methods obviously.

## Introduction

With the increasing popularity of major social media, people can express their attitude towards almost everything at any time through online websites, in the form of product reviews, blogs, twitters and microblogs. In recent years, automatic stance detection has attracted wide attention due to its wide applications, especially in the field of social media analysis, argument mining, truth finding and rumor detection [1, 2]. Stance detection is a basic study of text opinion mining, which usually has two key inputs: (1) a target and (2) a post or comment made by an author. Given two inputs, the purpose of stance detection is to analyze the stance tendency such as “favor, against or neutral” towards specific targets expressed in the text. The target can be an event, a policy, a social phenomenon, or a product [3].

So far, a considerable amount of literature has been published on stance detection [4–7]. Stance detection is essentially a task of text classification, in which information such as words and topics in the targets and user’s texts are used as features in traditional machine learning models, such as Logistic Regression, Naive Bayes, Decision Tree and Support Vector Machine [5, 8, 9]. In some advanced works, deep neural models such as Convolutional Neural Network (CNN), Recurrent Neural Network (RNN) and Long Short Term Memory (LSTM) have been used to learn the representation of targets and texts, and then perform text classification based on the representation [10–15]. Over the past two years, language pre-training models such as BERT [16], GPT [17], and XLNet [18] have brought remarkable progress. By pretraining on unlabeled corpus and fine-tuning on labeled ones, BERT-like models achieved state-of-the-art performance on many Natural Language Processing tasks. Among them, BERT has become an important part of various NLP models because of its effectiveness and universal usability. However, due to the large scale of the model, the practical application requires a lot of computing resources, and the time cost is high.

In the field of neural network optimization, it is an effective way to transform a large parameter model into a small parameter model to achieve faster inference speed and less computation [19]. It has been demonstrated that distilling knowledge from BERT can improve the performance of neural models [20–22]. Therefore, we propose a novel stance detection model BERTtoCNN based on knowledge distillation in this paper. BERTtoCNN uses the BERT model as the teacher model, and introduces the implicit knowledge learned from teacher model into the student model Text-CNN. Stance detection is different from ordinary sentiment analysis, and the learned representations should be as close as possible for texts with the same stance on a certain target. Inspired by Tung’s work [23] in the field of computer vision, we introduce the similarity-preserving loss combined with the classic distillation loss for further optimization, which uses the pairwise activation similarities within each input mini-batch to supervise the training of a student network with a trained teacher network. We conduct experiments and test the proposed method on the open Chinese and English stance detection datasets. The experimental results show that our model outperforms the competitive baseline methods significantly.

The main contributions of this paper can be summarized as follows:

- We propose a novel knowledge distillation model BERTtoCNN for the task of stance detection. In addition to the distillation loss, we introduce the similarity-preserving loss for further optimization. To the best of our knowledge, this is the first attempt that uses similarity-preserving loss in NLP tasks. At the same time, this is the first work combines the distillation loss and similarity-preserving loss in a joint knowledge distillation framework.
- We test with different settings of the BERTtoCNN and find that both classic distillation loss and similarity-preserving loss can help the stance detection task, BERTtoCNN further improves the performance by combining them together.
- We thoroughly investigate several baseline methods including recent neural models for comparison on the SemEval-2016 twitter stance detection and NLPCC-2016 microblog stance detection datasets. Experiment results show that our model outperforms various baseline methods.

## Preliminaries

### Transformer and BERT

**Fig 1:**
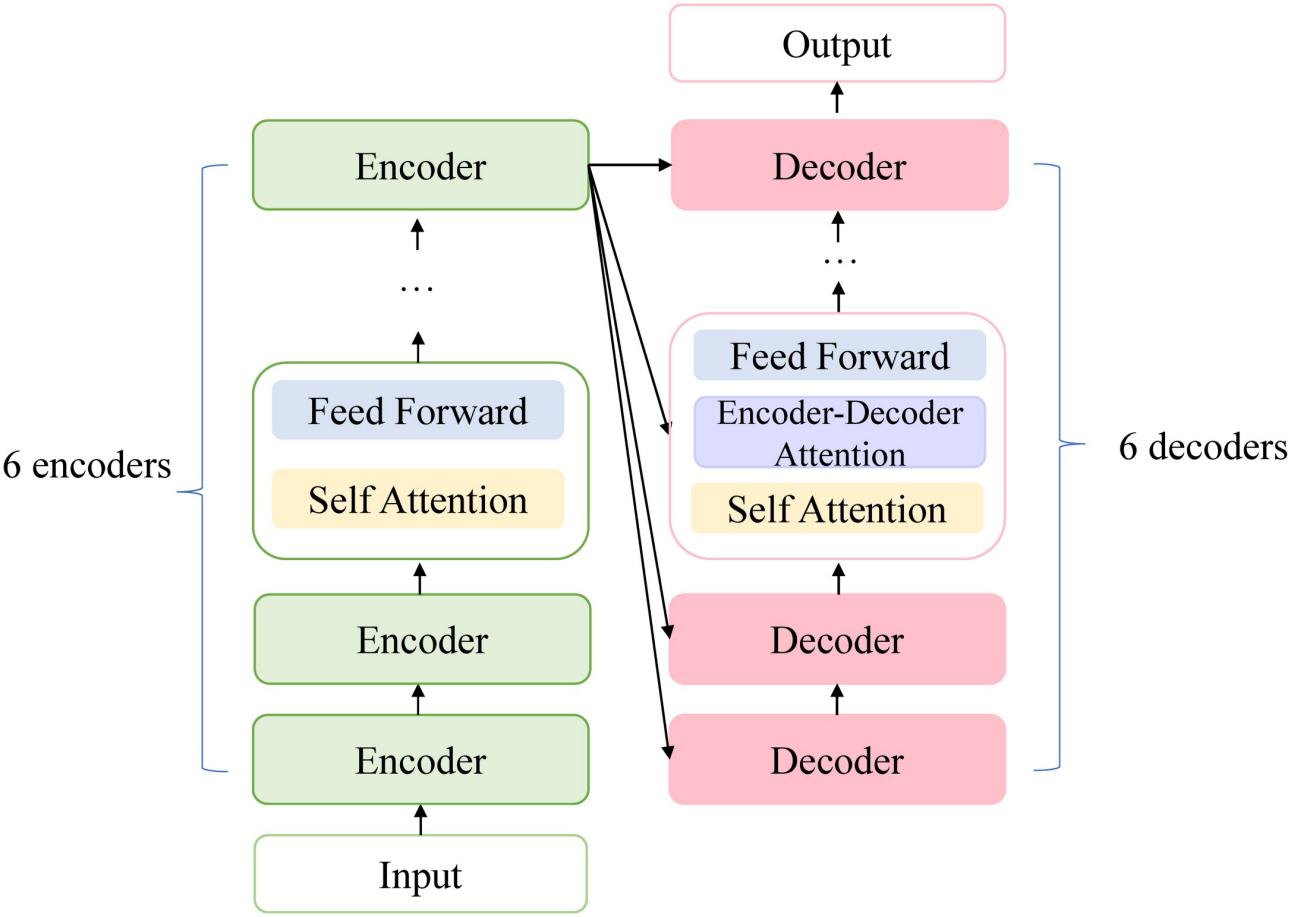
The transformer structure used in PLM.

Many recent pre-trained language models, such as BERT [16], XLNet [18] and RoBERTa [19], are built with Transformer [24] layers. Transformer is composed of 6 encoder-decoder superpositions, which are the same in structure, but do not share weights with each other. That is, one layer in the encoder corresponds to one layer in the decoder. After embedding, MHA (Multi-head Concern) and FFN (Fully Connected Feedforward Network) are followed, and there are residual connections between each sub-layers. The structure of transformer is shown in Fig 1.

BERT consists of multi-layer bidirectional transformers. Due to the self-attention mechanism, each word need to compute the attention with all the other words. Hence, the maximum path length between any two words can be controlled to be 1, which can capture the long-distance dependence.

To be more specific, BERT-base contains 110 M parameters by stacking twelve Transformer blocks, while BERT-large expands its size to even 24 layers. Obviously, the inference speed for these models would be much slower than classic architectures [25].

### Knowledge distillation

**Fig 2:**
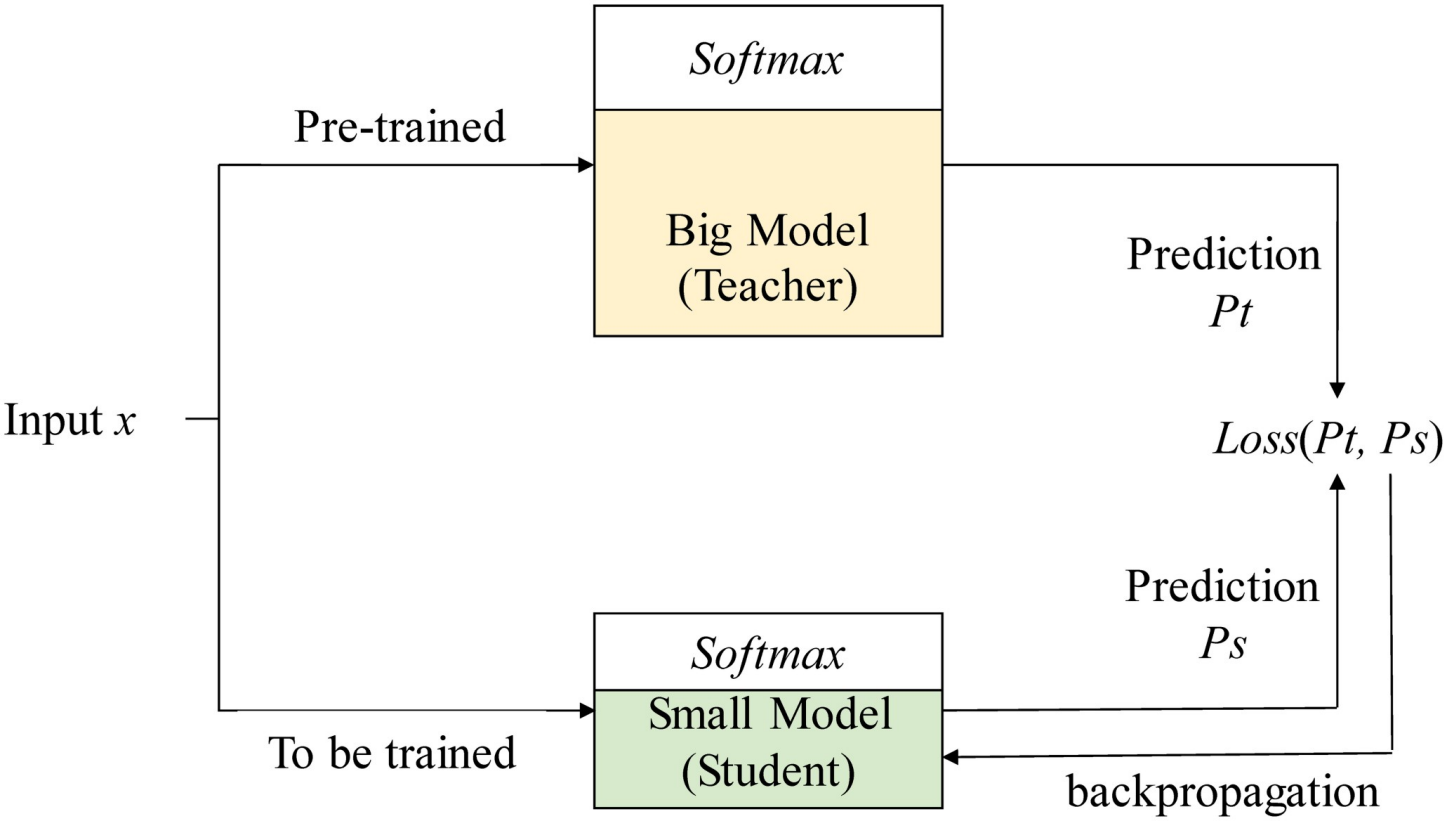
Classic knowledge distillation approach.

Knowledge distillation is a common model compression and transfer learning method. Through the “teacher” model and the “student” model, the implicit knowledge(dark knowledge) learned from the “teacher” model of complex network is **distilled** into the simple “student” model. So as to obtain higher generalization ability of “teacher” model, and the advantages of less storage space and faster inference are preserved at the same time. As shown in Fig 2, student model often uses an independent structure, whose effect however, depends mainly on the knowledge transferred from the teacher model. For the same input vector *x*, the larger teacher model generates a prediction *Pt* after training, and the lightweight student model generates a prediction *Ps* after training according to the knowledge transferred from the teacher. Both of them use a parameter *α* to calculate the loss *Loss(Pt, Ps)*.

## Model

**Fig 3:**
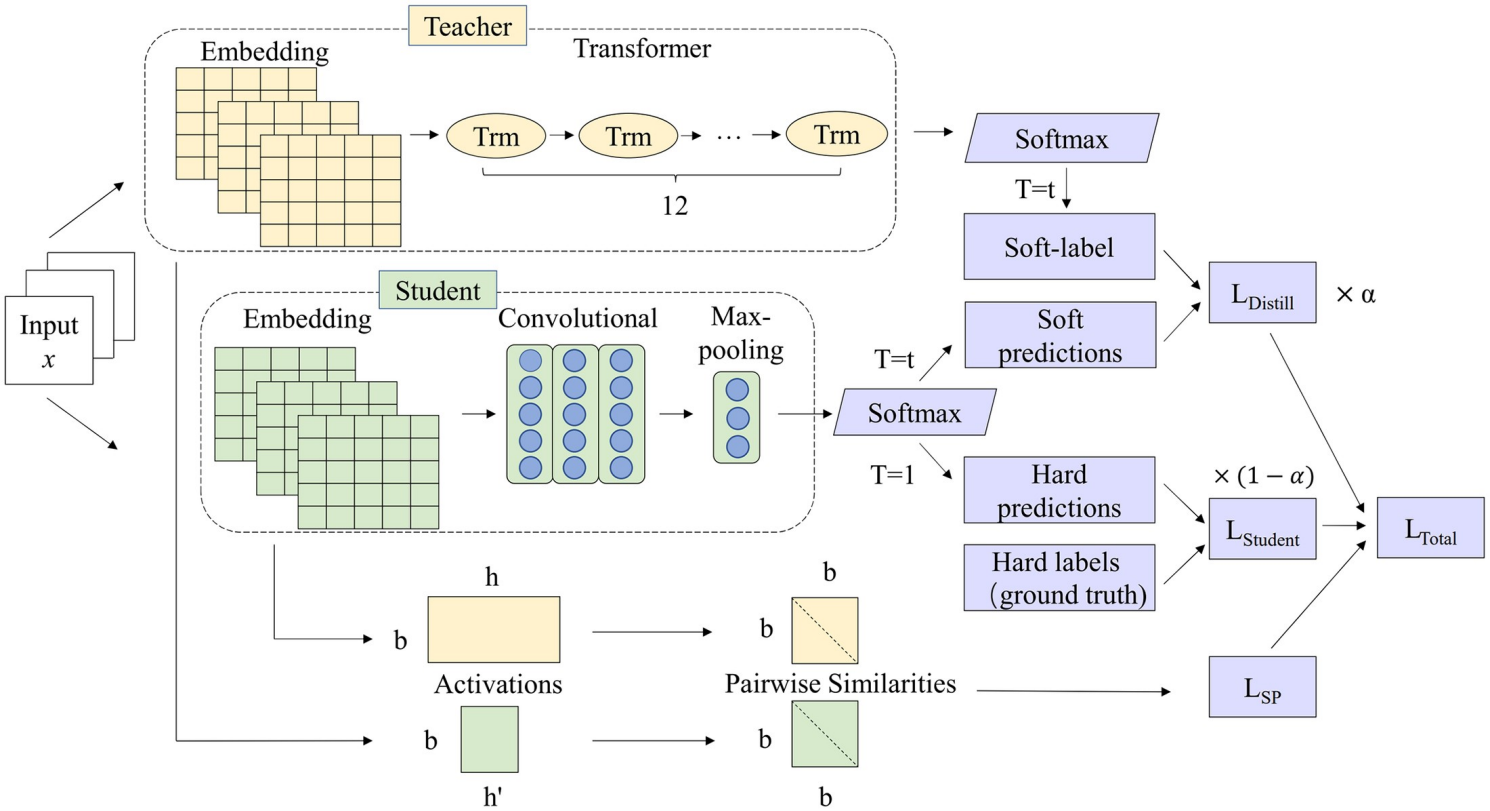
The structure of BERTtoCNN model.

In this section, we will present a novel knowledge distillation model BERTtoCNN for the task of stance detection. Given a text and a target, to predict the author’s stance towards the target, we would like to perform text classification based on the representation. BERTtoCNN model is shown in Fig 3, which consists of three parts: the BERT-Base model as the **Teacher model**, the Text-CNN model as the **Student model** and **Knowledge distillation**.

### Task definition

Let $D=\{x_{i}={\left(s_{i},t_{i},y_{i}\right)}_{i=1}^{N}\}$ be a dataset with *N* instances, each consisting of a text *s*_*i*_, a target *t*_*i*_, and a stance label *y*_*i*_. Each text is denoted as a word sequence *s*_*i*_ = { *w*_*i*0_, *w*_*i*1_, …, *w*_*in*_ }, and the target is denoted as a word sequence *t*_*i*_ = { *t*_*i*0_, *t*_*i*1_, …, *t*_*im*_ }, where *n* and *m* is the number of words in *s*_*i*_ and *t*_*i*_ respectively, and each word *w* ∈ *s_i_* ∪ *t_i_* belongs to the vacabulary set V. Given the instances with known stance labels, our goal is to predict the stance labels for “unknown” instances.

Now we come to the process of stance detection using BERTtoCNN model. First, we input the target and text into the BERT model at the same time. After training the BERT model, we save the output of last layer in the model, and further **soften** the output to obtain “soft label”. Then we train the student model Text-CNN based on the “knowledge” distilled from the BERT model. Finally, we perform stance detection using the student model.

### Teacher model

We use **BERT-Base** as the teacher model in BERTtoCNN. In this section, we will introduce how to apply **BERT-Base** for text stance detection and pass knowledge to the student model in detail.

#### Pre-training

In the pre-training step, the input vector of BERT consists of three parts:

- Token embedding, which is used to transform words into a limited set of common sub-word units. In this part, words are converted through WordPiece (word segmentation).
- Segment embedding, which is used to distinguish two input sentences, such as multiple input texts in multi-text classification. We fomulate the stance detection task as a sentence pair (text and target) task, as a result, segment embedding is needed.
- Position embedding, which encodes the position of words into feature vectors.

It should be noted that at the beginning of each text, a [CLS] symbol needs to be added, and the target and the post are separated by the [SEP] symbol.

#### Fine-tuning

After completing the pre-training process, it is necessary to perform fine-tuning for specific tasks. In the output hidden vector, we take the special mark [CLS] to construct the pooled output **h** of the final hidden state corresponding to the final hidden vector $C\in {\mathbb{R}}^{H}$. The parameter matrix of a classification network $W\in {\mathbb{R}}^{K*H}$ is passed to softmax layer to obtain the stance label *p*. This process can be formulated as follows:

$$\begin{array}{c} p=softmax(C\cdot W^{T}) \end{array}$$ (1)

### Student model

We use the BERT model to obtain the “soft label”, and pass it to the student model Text-CNN in BERTtoCNN. Next, we will introduce the student model.

#### Text-CNN

Convolutional Neural Network (CNN) is used for image recognition in the initial period. It has also achieved good results in Natural Language Processing tasks. The key reason we adopt Text-CNN as the student model is the way it uses local connections and shares weights. Weight reduction makes the network easy to optimize and prevents the risk of over-fitting. At the same time, Text-CNN has the advantage of parallel computing, which is similar to BERT.

Similar to the teacher model, we use the **BERT-Base** model to convert each word in the text and target into a one-dimensional vector, the output is the vector representation $e_{i}\in \mathbb{R}$^d^ of each input word *w*_*i*_. A convolutional filter is a list of linear layers with shared parameters. The input of the linear layer is the concatenation of word embeddings in a fixed-length window size *l*_*s*_, which is denoted as $I_{s}=[e_{i},e_{i+1},e_{i+l_{s}-1}]\in {\mathbb{R}}^{d\times l_{s}}$. We use a kernel $W_{s}\in {\mathbb{R}}^{d\times l_{s}}$ to perform convolution operation with the input *I*_*s*_ to generate a feature *c*_*i*_:

$$\begin{array}{c} c_{i}=f\left(W_{s}\cdot I_{s}+b_{s}\right) \end{array}$$ (2)

where *b*_*s*_ denotes the bias parameter, *f* is the non-linear function.

**Fig 4:**
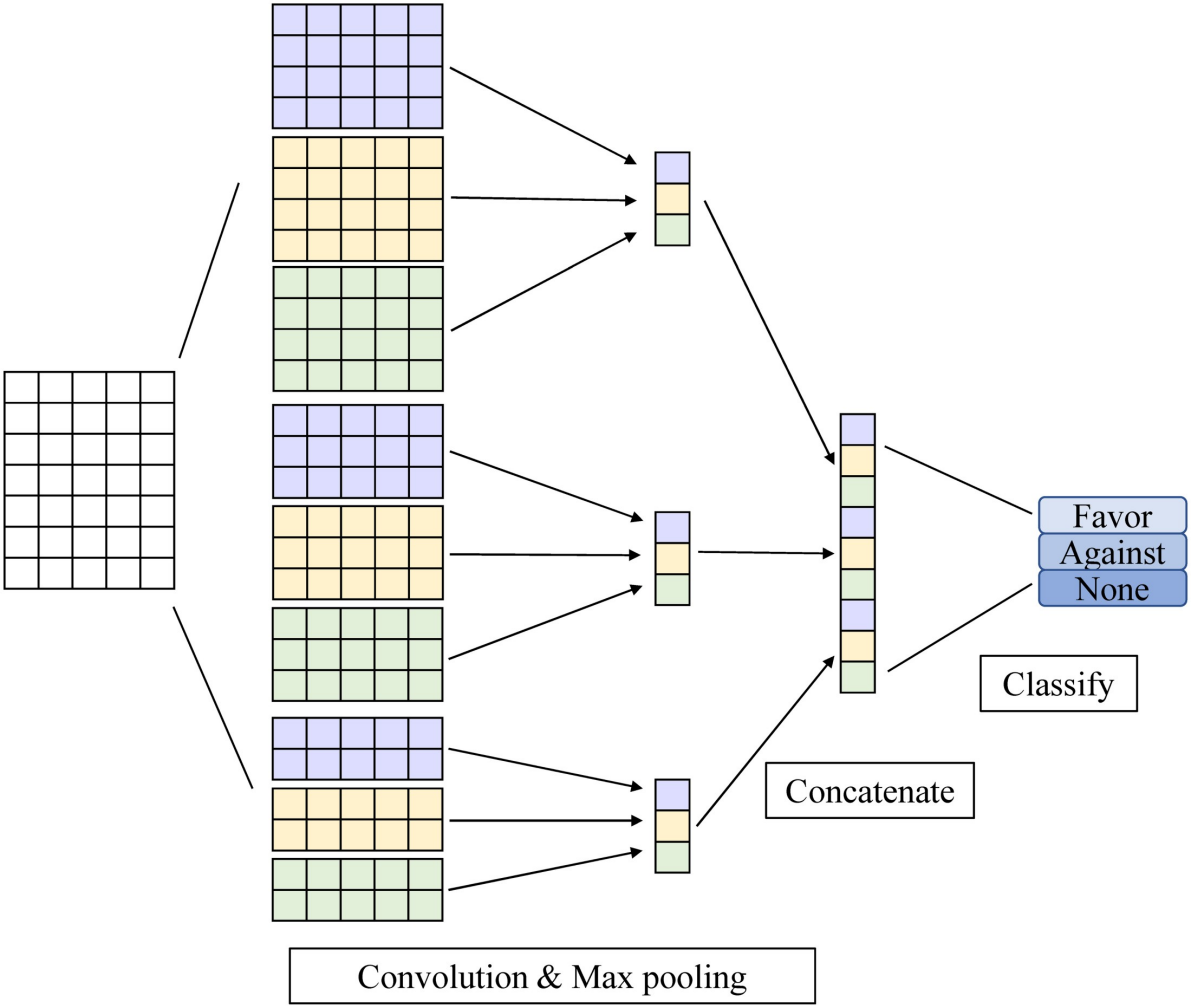
Text-CNN as the student model.

Then, we can concatenate them to obtain feature map *c* as the output of linear layer: *c* = [*c*_1_; *c*_2_; …; *c*_*n*−*l*+1_]. In order to capture the global semantics of a text, we feed the output of a convolutional filter to a max-pooling layer, resulting in an output vector with fixed length. Finally, it is used as the input of softmax layer, and the probability distribution of the stance label is obtained, which is called “soft prediction”. The structure of Text-CNN model is shown in Fig 4.

### Knowledge distillation

Given the teacher model and the student model, we will introduce the process of knowledge distillation in this section.

In order to learn knowledge from teacher model, now we only need to match the softmax distribution of the student model with the teacher under the given input, instead of matching the softmax distribution of the student model with the real label distribution. The teacher model provides the probability logits and estimated labels for the samples, and the student network learns from the teacher’s outputs.

#### Classic distillation loss

Usually, knowledge distillation is carried out at the output layer. The student model tries to imitate the behavior of teacher model given any data point. Inspired by Ba and Caruana [26], we use logits of teacher model as labels to train the student model, which contain more information than hard label.

Given the **logits z** of the output layer in a teacher model, *z*_*i*_ is the logits for the i-th class, the discrete probability output *p*_*i*_ corresponding to an input can be estimated by a softmax function:

$$\begin{array}{c} p_{i}=\frac{\text{exp}({\text{z}}_{\text{i}})}{\sum_{\text{j}}\text{exp}\left({\text{z}}_{\text{j}}\right)} \end{array}$$ (3)

The essence of adopting feature matching strategy in softmax layer is to use the output of softmax as supervision. In order to make the score vector softer, distillation temperature *T* is added in softmax layer to control the importance of each soft label as a divisor:

$$\begin{array}{c} p_{i}=\frac{\text{exp}({\text{z}}_{\text{i}}/T)}{\sum_{\text{j}}\text{exp}\left({\text{z}}_{\text{j}}/T\right)} \end{array}$$ (4)

By increasing *T*, the original probability distribution can be smoothed, and the similarity between data can be revealed better. While the knowledge distillation is not executed, the input to student model becomes the one-hot vector from the ground-truth transformation, which is called “hard target”. The soft label and ground truth label of teacher model are important to improve the performance of student model, and are used to extract **distill loss** and **student loss**, respectively.

Therefore, the classic distillation loss can be defined as follows:

$$\begin{array}{c} L_{Classic\_KD}=\alpha \cdot H\left(y,\sigma \left(z^{S}\right)\right)+\left(1-\alpha \right)\cdot T^{2}H(\left(\sigma \left(z^{T}/T\right),\sigma \left(z^{S}/T\right)\right) \end{array}$$ (5)

where *H*(.) is the cross entropy loss function, *σ*(.) is the softmax function, *T* is the temperature parameter, *y* is the one-hot vector indicating the ground truth class, **z**^*S*^ and **z**^*T*^ are the output logits of the student and teacher networks, respectively. In addition, *α* is the weighted hyper-parameter to balance the student loss and distill loss.

#### Similarity-preserving loss

If two inputs produce highly similar activation in the teacher network, it will help guide the training of the student network, and at the same time lead to highly similar activation of two inputs in the student network. Inspired by this observation, similarity-preserving loss is proposed [23].

Given an input mini-batch, we can define $Q_{T}^{\left(l\right)}\in R^{b\times h}$ as the activation map produced by the teacher network at a particular layer *l*, and $Q_{S}^{\left(l^{\prime }\right)}\in R^{b\times h^{\prime }}$ as the activation map produced by the student network at a corresponding layer *l*’, where *b* is the batch size, and *h* is spatial dimensions.

Given an input mini-batch of *b* texts, we compute pairwise similarity matrices from the output activation maps. The *b* × *b* matrices encode the similarities in the activation of the network as elicited by the texts in the mini-batch.

First, we have

$$\begin{array}{c} {\bar{G}}_{T}^{\left(l\right)}=Q_{T}^{\left(l\right)}\cdot {Q_{T}^{\left(l\right)}}^{⊤};G_{T}^{\left(l\right)}={\bar{G}}_{T}^{\left(l\right)}/{\left\|{\bar{G}}_{T}^{\left(l\right)}\right\|}_{2} \end{array}$$ (6)

where ${\bar{G}}_{T}^{\left(l\right)}$ is a *b* × *b* matrix. Intuitively, we can apply a row-wise L2 normalization to obtain $G_{T}^{\left(l\right)}$.

Similarly, for student model, we have:

$$\begin{array}{c} {\bar{G}}_{S}^{\left(l^{\prime }\right)}=Q_{S}^{\left(l^{\prime }\right)}\cdot {Q_{S}^{\left(l^{\prime }\right)}}^{⊤};G_{S}^{\left(l^{\prime }\right)}={\bar{G}}_{S}^{\left(l^{\prime }\right)}/{\left\|{\bar{G}}_{S}^{\left(l^{\prime }\right)}\right\|}_{2} \end{array}$$ (7)

After that, the similarity-preserving loss can be defined as:

$$\begin{array}{c} L_{SP\_KD}\left(G_{T},G_{S}\right)=\frac{1}{b^{2}}\sum_{(l,l^{\prime })\varepsilon L}{\left\|G_{T}^{\left(l\right)}-G_{S}^{\left(l^{\prime }\right)}\right\|}_{F}^{2} \end{array}$$ (8)

where the ‖⋅‖_*F*_ means the Frobenius norm. Eq 8 is a summation overall (*l*, *l*′) pairs of the mean element-wise squared difference between the $G_{T}^{\left(l\right)}$ and $G_{S}^{\left(l^{\prime }\right)}$ matrices.

#### Objective function

The overall objective function consists of two parts: the classic distillation loss and the similarity-preserving loss. We use a hyper-parameter to balance the two parts. Given Eqs 5 and 8, the objective function of BERTtoCNN can be defined as follows:

$$\begin{array}{c} L_{KD}=\gamma L_{Classic\_KD}+\left(1-\gamma \right)L_{SP\_KD}\left(G_{T},G_{S}\right) \end{array}$$ (9)

where *γ* is a balancing hyperparameter for the classic distillation loss and the similarity-preserving loss. In the training process of the model, we try to minimize the objective function by using the Adam optimizer [27].

#### Data argumentation

In the distillation approach, a small dataset may not be enough for the teacher model to express its knowledge completely. In order to make up for the lack of data in the English Twitter corpus and obtain a larger data sets, we use the EDA: Easy Data Augmentation method [28] to expand the dataset. We use synonym replacement, random insertion, random swap and random deletion to process twitter text.

- Synonym Replacement (SR): It refers to randomly selecting *n* non-stop words from a sentence and replacing each of these words with one of the randomly selected synonyms.
- Random Insertion (RI): Randomly select a non-stop word in the sentence and insert one of its synonyms into any position in the sentence. Repeat it for *n* times.
- Random Swap (RS): Swap the positions of two random words in a sentence. Repeat it for *n* times.
- Random Deletion (RD): Randomly delete each word in the sentence with the probability of *p*.

EDA mainly defines three parameters: *n* representing the number of words modified in a sentence, *β* representing the proportion of words modified in a sentence, and *n*_*aug* representing the number of new sentences generated by a sentence. We vary the value of *n* for SR, RI, and RS based on the sentence length *l* with the formula *n* = *β* × *l*. For Random Deletion (RD), *p* = *β* × *n_aug*.

Table 1 shows the data augmentation results of target “*Climate Change is Concern*” in English twitter stance detection corpus.

**Table 1:** The EDA result of Climate Change is Concern.

Post	Method
I’m gonna side with the really smart and well educated folks on this one guys	No-ops
I’m gonna side with the really smart and well educated folks on this one along guys	RI
I’m gonna side with the really chic and well educated folks on this one guys	SR
I’m gonna side with well really smart and the educated folks on this one guys	RD
I’m gonna side the with really smart and well educated folks on this one guys	RS

## Experiments

We apply the proposed model BERTtoCNN to the task of text stance detection to evaluate the performance. In this section, we design experiments to answer the following research questions: (i) Does the BERTtoCNN model perform better than other baseline methods? (ii) How much can knowledge distillation help for stance detection compared with traditional neural models? (iii) Does the introduction of similarity-preserving loss help on top of classic distillation loss for this task?

### Dataset

We use two text stance detection datasets to perform the experiments. One is the SemEval-2016 task 6 twitter stance detection dataset [29], the other is the NLPCC-ICCPOL-2016 task 4 Chinese microblog stance detection dataset [3]. Each data in the datasets is represented in the format of triples (“stance”,“target”,“text”), where “stance” labels including **Favor**, **Against** and **None**. The statistics of the dataset is illustrated in Table 2.

**Table 2:** The SemEval-2016 stance dataset.

	%of instances in Train					%of instances in Test			
Target	#total	#train	Favor	Against	None	#test	Favor	Against	None
Atheism	733	513	17.9	59.3	22.8	220	14.5	72.7	12.7
Climate Change	564	395	53.7	3.8	42.5	169	72.8	6.5	20.7
Feminist Movement	949	949	31.6	31.6	49.4	285	20.4	64.2	15.4
Hillary Clinton	983	689	17.1	57.0	25.8	295	15.3	58.3	26.4
Legal. Abortion	933	653	18.5	54.4	27.1	280	16.4	67.5	16.1
Total	4163	2914	25.8	47.9	26.3	1249	23.1	51.8	25.1
**URL**	https://alt.qcri.org/semeval2016/task6/								

For the English dataset, the training set contains 2,914 English tweets with stance labels, and 1,249 tweets in test set. There are 5 targets: “Atheism”, “Climate Change is Concern(**CC**)”, “Feminist Movement(**FM**)”, “Hillary Clinton(**HC**)” and “Legalization of Abortion(**LA**)”.

The Chinese dataset contains 4,000 Chinese microblogs with stance labels, among which 3,000 microblogs are for training and 1,000 microblogs for testing. There are also 5 targets: “IPhone SE”, “Set off firecrackers in the Spring Festival(**SF**)”, “Russian anti-terrorism operations in Syria(**RA**)”, “Two child policy(**TP**)” and “Prohibition of motorcycles and restrictions on electric vehicles in Shenzhen(**PM**)”. The statistics of the dataset is illustrated in Table 3.

**Table 3:** NLPCC-ICCPOL-2016 stance dataset.

	%of instances in Train					%of instances in Test			
Target	#total	#train	Favor	Against	None	#test	Favor	Against	None
IphoneSE	800	600	40.8	34.8	24.4	200	37.5	52.0	10.5
Set off firecrackers in the Spring Festival	800	600	41.7	41.7	13.6	200	44.0	47.0	9.0
Russia’s anti terrorist operations in Syria	800	600	41.7	41.7	13.6	200	47.0	43.0	10.0
Two child policy	800	600	43.3	33.3	23.4	200	49.5	47.5	3.0
Prohibition of motorcycles and restrictions on electric vehicles in Shenzhen	800	600	26.7	50.0	23.4	200	31.5	55.0	13.5
Total	4000	3000	38.8	40.3	20.9	1000	41.9	48.9	9.2
**URL**	http://tcci.ccf.org.cn/conference/2016/pages/page05_evadata.html								

### Experimental settings

#### Baseline methods

For the English Twitter stance detection dataset, we consider the following baseline methods for comparison.

- MIRTE: The model [11] uses two Recurrent Neural Network (RNN) classifiers. The first RNN is used to learn features through distant supervision of two large unlabeled data sets during initialization. The second RNN is the classifier. It uses the word2vec model to train the embedding of words and phrases, and then uses these features to learn sentence representations for stance detection.
- DC-BLSTM: Based on Siddiqua’s work [30], Yang et al. [31] proposed a two-stage deep attention neural network(TDAN) for target-specific stance detection. This model employs densely connected BI-LSTM to encode tweet tokens and traditional bidirectional LSTM to encode target tokens. They decompose the ternary classification problem into two binary classification problems to mitigating the imbalanced distribution of labels.

And we choose the following methods as comparative baselines on the Chinese microblog stance detection dataset.

- RUC MMC: Dian et al. [5] used five manually selected features as input of Random Forest and SVM model. They achieved the best results in NLPCC-ICCPOL 2016 task 4.
- ATA: A two-stage attention model proposed by Yue [4]. Firstly, the attention mechanism is applied to model target, then the context is matched with the target representation to obtain attention signal, and finally, the target specific text representation for stance classification is formed.

#### Evaluating metrics

We use Precision(*p*), Recall(*r*) and F-score as the evaluation metrics, which is similar to previous work [3]. Precision(*p*) is the proportion of correctly classified positive samples predicted by the classifier to be positive samples, and Recall(*r*) refers to the proportion of correctly classified positive samples in the true number of positive samples. F-score is the harmonic average value of *p* and *r*.

$$\begin{array}{c} F_{Favor}=\frac{2*P_{Favor}*R_{Favor}}{P_{Favor}+R_{Favor}} \end{array}$$ (10)

$$\begin{array}{c} F_{Against}=\frac{2*P_{Against}*R_{Against}}{P_{Against}+R_{Against}} \end{array}$$ (11)

After calculating the *F*_*Favor*_ and *F*_*Against*_, respectively, we can average the two to obtain the *F*_*Avg*_ as the final result.

$$\begin{array}{c} F_{Avg}=\frac{F_{Favor}+F_{Against}}{2} \end{array}$$ (12)

#### Experimental setup

We perform the stance detection experiments according to the following steps. We first train our model on training data, and save the model which has the best performance. The Chinese BERT pre-trained model “**BERT-Base, Chinese**” and the English BERT pre-trained model “**BERT-Base, Uncased**” released by Google are used as teacher models. The models use 12-layer of transformer, output 768 dimension vectors, the head number of multi-head attention is 12. The total number of trainable parameters of the two BERT models above are the same (110M). For our model, the teacher model and the student model use the same settings, in which the learning rate is set to 1e-5 and the batch size is set to 8. As for the hyper-parameters *α*, *γ* and *T*, we choose 0.5, 0.5 and 60 respectively. We will conduct further parameter sensitivity experiment for *α* and *T* later. We run the model for several iterations until convergence.

For all other baseline methods, we directly get the results reported in their papers, because we conduct experiments based on the same dataset and the same settings.

### Experimental results

#### Comparison to other methods

In this part, we compare the F-score of our model with the baselines. Note that the experimental results are all obtained without data augmentation(EDA). From the results in Table 4, we can draw the following:

1. Our model BERTtoCNN significantly outperforms the three baseline methods in both two stance detection tasks. The improvements in Chinese task are more obvious.
2. Compared with other baselines (including traditional machine learning methods and deep neural models), BERT is effective in learning semantics, and achieves the best result. Our model BERTtoCNN transfers knowledge from BERT model, and obtains comparable results with BERT, which is much faster than BERT in running speed.
3. By comparing with TextCNN, the average F-score of BERTtoCNN is higher than that of TextCNN. It shows that BERTtoCNN can learn useful information from teacher model to help improve the performance of student model.

**Table 4:** The performance of BERTtoCNN compared with the baseline methods.

	Model	*F_Favor_*	*F_Against_*	*F_avg_*
**English**	MIRTE	0.593	0.763	0.678
	DC-BLSTM	0.571	0.691	0.632
	Text-CNN	0.649	0.696	0.673
	BERT	0.670	0.694	0.682
	**BERTtoCNN**	**0.660**	**0.695**	**0.678**
**Chinese**	RUC MMC	0.697	0.724	0.711
	ATA	0.762	0.671	0.717
	Text-CNN	0.688	0.702	0.695
	BERT	0.741	0.755	0.748
	**BERTtoCNN**	**0.733**	**0.749**	**0.741**

All the above results demonstrate that our method can reduce the training cost and achieve considerable performance at the same time.

#### Parameter sensitivity analysis

In order to analyze the influence of hyper-parameters, we conduct two parameter sensitivity experiments on English twitter stance detection dataset.

**Fig 5:**
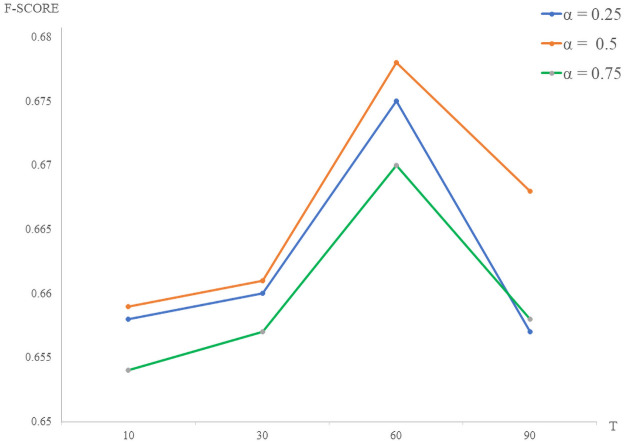
F-score curves with the variation of parameter *α* and *T*. Among them, *α* is the parameter of balancing the soft and hard labels in the knowledge distillation loss, and *T* is the temperature, which is used to adjust the smoothness of prediction distribution in knowledge distillation.

- **Effects of hyperparameter *α* and *T***
  In BERTtoCNN, the hyper-parameter *α* controls the loss rate of “soft label” and “hard label”. We set *α* to 0.25, 0.5 and 0.75, similar to the settings in previous work [32]. From Fig 5, we find that with the increase of *α*, the F-score also increases correspondingly. When *α* is 0.5, F-score reaches its highest point, and it decreases when *α* is larger than 0.5. The results demonstrate that the increase of *α* can improve the result of stance detection. On the one hand, a larger *α* can force the student model to learn more knowledge from teacher model. On the other hand, experiments show that soft label plays a role of regularization, which makes the convergence of student model more stable.
  The hyper-parameter *T* mainly controls the smoothness of prediction distribution. In this experiment, we set the adjustment space of *T* is {10, 30, 60, 90}, similar to the settings in previous work [21]. Fig 5 shows that when *T* increases from 10 to 60, the performance of the F-score is improved. When *T* is equal to 60, BERTtoCNN achieves the best performance. The overall results in Fig 5 show that the increase of *T* can improve the results of stance detection, which reflects the stronger generalization ability of teacher models. However, over-generalization will also have a negative impact on the classification results.
- **Effects of hyper-parameter *γ***
  Note that *γ* is a weight parameter for balancing the classic distillation loss *L*_*Classic*_*KD*_ and similarity-preserving loss *L*_*SP*_*KD*_. In other words, the larger *γ* is, the more student network will learn from the similar activation in the teacher network. That is to say, the greater *γ* is, the greater the effect of similarity loss is. We set *γ* to 1, 0.5, 0.1, 0.01 and 0. From Table 5, we find the model achieves the best performance when *γ* is 0.5. We found that the similarity-preserving loss brought about an improvement of 4.1% on F-score for our model (*γ* = 0, without similarity-preserving loss). When *γ* gradually decreases from 0.5 to 0, the improvement of classification results becomes smaller and smaller. This proves that the introduction of similarity-preserving loss, can learn the useful feature information between students, so as to improve the classification results.

**Table 5:** F-score with the variation of hyperparameter *γ*.

	*γ* = 1	*γ* = 0.5	*γ* = 0.1	*γ* = 0.01	*γ* = 0
F-score	0.671	0.678	0.670	0.658	0.651

#### Effects of data argumentation

In this section, we explore the impact of different size of dataset on the classification results. The original English twitter stance detection dataset contains only 2,914 tweets in the training set and 1,249 tweets in the test set. As a result, the classification effect is limited.

We adopt the EDA data augmentation method (as described in Section *Data argumentation*) to expand the dataset for improving the classification results. Note that, the parameter *n* represents the number of words modified in a sentence, *β* stands for the percentage of words enhanced in each sentence, and *n*_*aug* is the number of new sentences generated by a sentence. We let *n*_*aug* as 16, 8 and 4, and *β* as 0.05 and 1 for BERTtoCNN to compare the results. As mentioned above, the best results of BERTtoCNN are obtained when *T* is 60 and *α* is 0.5, so we also take these two values in this experiment.

The experimental results are shown in Table 6. We can see that the best results are obtained when *n*_*aug* is 16 and *β* is 0.1, and the F-score of the student model is 68.9%. Therefore, it can be concluded that with the increase of data sets, the performance of BERTtoCNN is improved.

**Table 6:** The comparison of EDA data and original data on the model. Within, we set n_aug = 1 represents the result of the original data without EDA on the model.

	*n*_*aug* = 1	*n*_*aug* = 4	*n*_*aug* = 8	*n*_*aug* = 16
*β* = 0.05	0.678	0.684	0.687	0.688
*β* = 0.1	0.678	0.682	0.687	0.689

## Related work

We compare and relate our work with the recent two lines of works, including stance detection and knowledge distillation with BERT.

### Stance detection

So far, a considerable amount of literature has been published on text stance detection, the methods adopted by researchers can be roughly divided into two categories: traditional machine learning based methods and deep learning based methods.

Traditional machine learning based methods focus on how to select features for the classification model. Besides simple textual features like bag-of-words (BoW), Xu et al. [33] used para2vec, LDA and LSA to represent the semantic information in tweets, and compared the results of different machine learning algorithms such as random forest and support vector machine (SVM). Javid et al. [34] integrated sentiment polarity into target and stance, and modeled the interaction of target, stance label and sentiment words in a probabilistic graph model. Dian et al. [5] used the combination of BoW model of synonym dictionary, character vector and word vector as features. They used SVM, random forest and decision tree for stance detection respectively, and finally merged these models.

Deep learning based methods for stance detection make attempts to learn the representations of target and text, and then perform text classification based on the representations. Early stage of deep learning method, Augenstein et al. [13] proposed a neural network architecture based on conditional encoding. A LSTM network is used to encode the target, followed by a second LSTM that encodes the tweet using the encoding of the target as its initial state. Experimental results showed that the model performed better than coding tweets and targets separately, which is consistent with the work of Luo et al. [35] and Du et al. [36]. With the introduction of attention mechanism, Bai et al. [7] proposed a BiLSTM-CNN model based with attention mechanism to focus on the target and text respectively. The BiLSTM and CNN model are used to obtain the text representation vector and local convolution features respectively. Then, these two features are used for classification.

When BERT and other pre-training language models are on the stage of deep learning, many scholars use BERT for stance detection. For example, Wang et al [6] proposed a stance detection model BERT-condition-CNN. They use BERT pre-trained model to obtain the representation vector of the text, and the relationship matrix condition layer between the targets and the text vector is constructed. Finally, CNN was used to extract the features of the condition layer to perform the classification.

In recent years, some scholars have began to explore how to use external common sense knowledge to enrich the feature representation in stance detection [37, 38]. With the integration of external knowledge, the results of stance detection will be improved. At the same time, the complexity of the model will be increased, and knowledge distillation will become more important.

### Knowledge distillation with BERT

The concept of knowledge distillation (dark knowledge extraction) was proposed by Hinton et al. [19] for the first time. By introducing “soft label” learn from teacher network as a part of loss function, knowledge distillation can induce the training of student network and realize knowledge transfer.

DistilBERT [22] first combined the idea of knowledge transfer with BERT. The model is similar to the BERT model, but DistilBERT has only 6 layers, while the BERT-Base has 12 layers. With the decrease of parameters and the number of layers, DistilBERT can still keep good performance. Jiao et al. [20] put forward a two-stage learning framework TinyBERT, which performed distillation in the pre-trained and task-specific learning stages respectively, so as to acquire general knowledge from teacher model and task-specific knowledge. In the task of GLUE, the result is equivalent to BERT (decreased by 3 percentage points), and the size of the model size is only 13.3% of that of BERT’s, and the inference speed is 9.4 times of BERT. Hou et al. [39] proposed DynaBERT for training sub-networks of different sizes. The first step is the training of width adaptation. Firstly, rewiring mechanism can be used to sort attention heads and neurons, and got a tailored teacher model, which can be used to initialize the student model. Then, different sizes of sub-networks are obtained as student models. The second step is to carry out width and depth adaptive training. The performance of this model ouperforms many similar compression models.

Although BERT-based distillation models have achieved good results in some NLP tasks, it has not been proved in the task of text stance detection. Text stance detection is different from ordinary sentiment analysis, and the learned representations should be as close as possible for texts with the same stance on a certain target. Therefore, in addition to the distillation loss, we introduce the similarity-preserving loss, which guides the training of a student network, so that input pairs with similar (dissimilar) activation in the teacher network have similar (dissimilar) activation in the student network. To the best of our knowledge, this is the first attempt that uses similarity-preserving loss in this task. At the same time, this is the first work combines the distillation loss and similarity-preserving loss in a joint knowledge distillation framework.

## Conclusion

In this paper, we propose a text stance detection model BERTtoCNN based on similarity-preserving knowledge distillation. In addition to the distillation loss, BERTtoCNN introduces the similarity-preserving loss for further optimization. To the best of our knowledge, this is the first work that combines the classic distillation loss and similarity-preserving loss in a joint knowledge distillation framework. We test with different settings of the BERTtoCNN and find that both classic distillation loss and similarity-preserving loss can help the stance detection task, BERTtoCNN further improves the performance by combining them together. Specifically, by comparing BERTtoCNN with other competative baseline methods, we find that pre-trained language models like BERT are effective in this task. By distilling the knowledge of the pre-trained model BERT, BERTtoCNN achieves comparable results, but improves the running time to a great extent. Finally, we perform data augmentation on the English twitter stance detection dataset, which proves that a larger dataset is more beneficial to improve the results.

There are a few directions we would like to explore in the future. First, the current work does not consider using other types of data in the text for stance detection. In the future, multimodal data (such as pictures and videos) can be incorporated into the model. Second, we will consider how to use other related tasks (such as text entailment) to help stance detection in the future. Finally, the annotation data acquisition of text stance detection has always been a very concerned problem. We will explore the text stance detection in the case of less labeled data, such as few shot learning. All these issues will be left as our future works.
